# Precision in robotic rectal surgery using the da Vinci Xi system and integrated table motion, a technical note

**DOI:** 10.1007/s11701-017-0752-7

**Published:** 2017-09-15

**Authors:** Sofoklis Panteleimonitis, Mick Harper, Stuart Hall, Nuno Figueiredo, Tahseen Qureshi, Amjad Parvaiz

**Affiliations:** 1 School of Health Sciences and Social Work, University of Portsmouth, James Watson West, 2 King Richard 1st Road, Portsmouth, PO1 2FR UK; 2 Poole Hospital NHS Trust, Longfleet Road, Poole, BH15 2JB UK; 3 Champalimaud Foundation, Av. Brasilia, 1400-038 Lisbon, Portugal; 4 Bournemouth University School of Health and Social Care, Bournemouth, UK

**Keywords:** Robotic surgery, Rectal surgery, Rectal cancer, da Vinci Xi, Table motion

## Abstract

Robotic rectal surgery is becoming increasingly more popular among colorectal surgeons. However, time spent on robotic platform docking, arm clashing and undocking of the platform during the procedure are factors that surgeons often find cumbersome and time consuming. The newest surgical platform, the da Vinci Xi, coupled with integrated table motion can help to overcome these problems. This technical note aims to describe a standardised operative technique of single docking robotic rectal surgery using the da Vinci Xi system and integrated table motion. A stepwise approach of the da Vinci docking process and surgical technique is described accompanied by an intra-operative video that demonstrates this technique. We also present data collected from a prospectively maintained database. 33 consecutive rectal cancer patients (24 male, 9 female) received robotic rectal surgery with the da Vinci Xi during the preparation of this technical note. 29 (88%) patients had anterior resections, and four (12%) had abdominoperineal excisions. There were no conversions, no anastomotic leaks and no mortality. Median operation time was 331 (249–372) min, blood loss 20 (20–45) mls and length of stay 6.5 (4–8) days. 30-day readmission rate and re-operation rates were 3% (*n* = 1). This standardised technique of single docking robotic rectal surgery with the da Vinci Xi is safe, feasible and reproducible. The technological advances of the new robotic system facilitate the totally robotic single docking approach.

**Electronic supplementary material:**

The online version of this article (doi:10.1007/s11701-017-0752-7) contains supplementary material, which is available to authorized users.

## Introduction

The increasing adoption of robotic rectal surgery is evident from the growing number of studies published on the subject [1–3]. This might be due to robotic systems offering superior three-dimensional views and advanced instrument ergonomics, thus enabling precise dissection in narrow spaces such as the pelvis [4, 5]. However, with previous systems (S & Si) certain technical challenges have arguably slowed down its wholesale adoption. Besides increased costs, colorectal surgeon’s using robotic systems have found the docking process lengthy and complex, complained of persistent arm clashing of the robot and the need to undock the robotic cart when moving from one operative quadrant to another. The da Vinci Xi® is the latest surgical platform developed by Intuitive Surgical and comes with several technological advances to address these issues.

We have developed a standardised technique for single docking robotic rectal surgery using the da Vinci Xi system, primarily based on a previously described laparoscopic technique [6] also applied on the da Vinci Si® [7]. In this technical note, we describe the technique using Xi system with integrated table motion and present an intra-operative video demonstrating the procedure steps.

## Surgical technique

In the accompanying video, we demonstrate a single docking totally robotic anterior resection on a 69-year-old male patient with a mid-rectal tumour. Pre-operative CT and MRI suggested a T3N0M0 rectal carcinoma. Following MDT discussion, decision was made to offer him surgery. The operative steps described below are demonstrated on the video.

### Patient positioning and port placement

The patient is placed on a vacuum bean mattress, supine in a modified Lloyd Davies position with the arms wrapped besides the body.

**Fig. 1:**
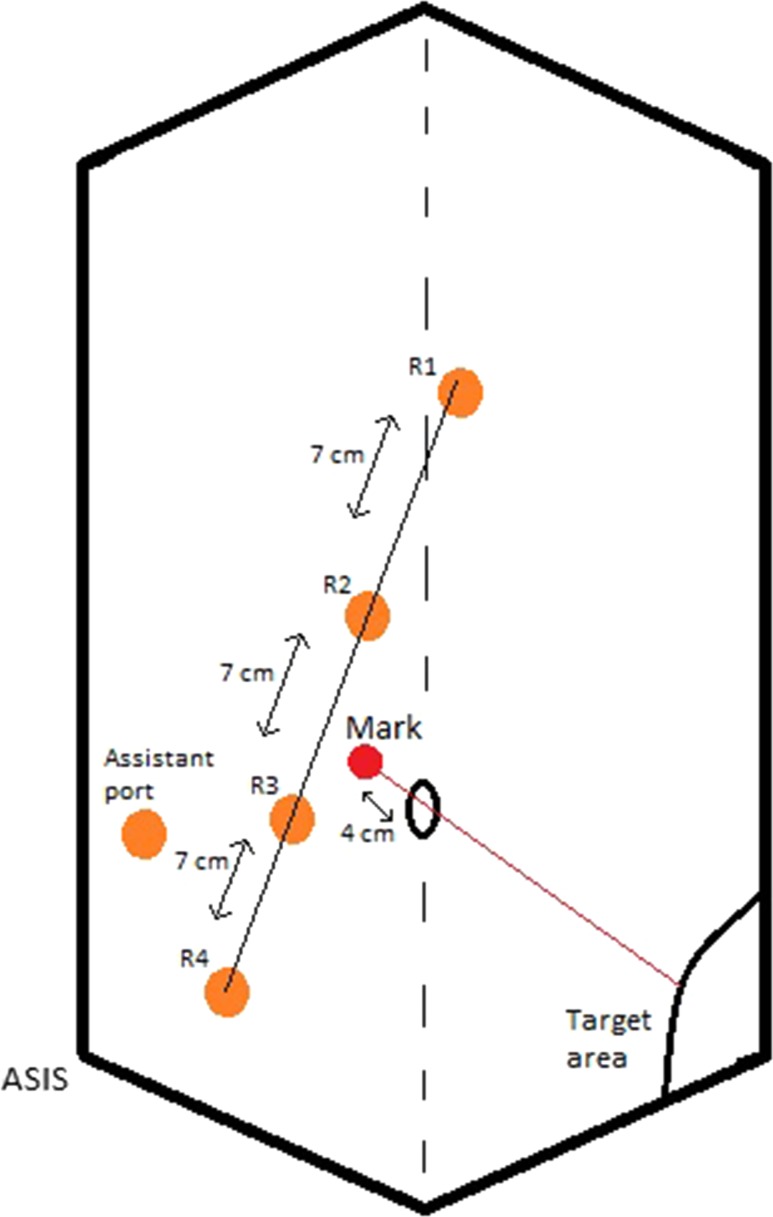
Port positions. Ports R1–R4 are placed on a straight line 7 cm apart. A mark is drawn 4 cm from the umbilicus on a straight line from the target area towards the umbilicus. The straight line where the ports are inserted needs to be lateral to this mark

Port configuration is demonstrated in Fig. 1. Port positioning is drawn on the patient’s abdomen after creating pneumoperitoneum and the robotic ports are inserted under direct vision. Ports R1–R4 are all placed 7 cm apart from each other in a straight line on the right side of the abdomen oblique to the midline. A 12 mm port is used for R4 and 8 mm ports for R1–R3. R4 is placed approximately 2 in. superior and medial to the right anterior superior iliac spine. An assistant 10-mm port is placed between and behind ports R3 and R4 for suction/irrigation, vessel ligation and retraction. The umbilical port site used to create pneumoperitoneum is closed once the robotic and assistant ports are inserted.

### Robot docking

Before docking commences the small bowel, omentum and transverse colon are displaced cranially. The robotic patient cart is set to the left lower abdomen and pelvis anatomy setting and brought towards the patient from the patients left side. Thereafter, a laser guided system displaying a green target is projected from the cart’s overhead boom, which is aligned to camera port (R2). The camera is then inserted in R2, pointed towards the rectosigmoid junction and selected as the target anatomy. The cart then automatically positions its boom in an optimised configuration to perform low anterior resection surgery. The remaining robotic arms are docked and the rest of the instruments inserted; traditionally with fenestrated bipolar forceps in R1, scissors with monopolar diathermy in R3 and Cadiere forceps in R4.

### Left colonic and splenic flexure mobilisation

The mesocolon is dissected medial to lateral and the inferior mesenteric artery is isolated and ligated 1 cm from its origin by applying disposable locking clips (Hem-o-lok^®^). Dissection continues laterally towards the abdominal wall and superiorly towards the spleen. The inferior mesenteric vein is divided at the lower border of the pancreas. A previously described standardised three-step approach is used for splenic flexure mobilisation [8]. Step one involves dissection over the lower border of the pancreas aiming to enter the lesser sac. Step two involves lateral colonic mobilisation towards the splenic flexure up to the splenocolic ligament. Before the final step commences the integrated table motion is used and the patient is repositioned from the Trendelenburg to the reverse Trendelenburg position. The reverse Trendelenburg helps with the final step of the splenic flexure mobilisation particularly in male patients with high BMI as it helps to displace the transverse colon downwards which helps to achieve separation of omentum from transverse colon. In step three, the omentum is separated from the transverse colon and the lesser sac is entered from above.

### Total mesorectal excision

For the total mesorectal excision (TME), the patient is positioned into the Trendelenburg position to move the small bowel out of the pelvis. Dissection commences posteriorly and proceeds in a stepwise manner as previously described [6] whilst great care is maintained during the lateral dissection to protect the hypogastric nerve plexus. During the last 5 cm of rectal mobilisation, a 30° scope looking upwards provides an additional view to ensure safe division of the anococcygeal ligament and achieve full mobilisation of the rectal tube. This step combined with the use of the EndoWrist robotic stapler greatly enhances sphincter preservation for low rectal cancers.

A robotic EndoWrist Stapler 45 mm® is attached to arm 4 and used to divide the rectum. The cart is then undocked and the specimen is extracted through a 4–5 cm incision using a wound protector. A circular stapler (CDH29 mm™) is used to perform the colorectal anastomosis. A flexible endoscope is routinely used to check the integrity of the anastomosis. Finally, a 20-mm drain is inserted into the pelvis and a defunctioning loop ileostomy is routinely performed for all patients with mid- or low-rectal tumours.

## Results

Thirty-three consecutive patients with rectal cancer underwent robotic rectal surgery with the da Vinci Xi system between November 2015 and December 2016. Their baseline characteristics are shown in Table 1.

**Table 1:** Baseline characteristics

	Total (*n* = 33)
Sex	
Male	24 (73%)
Female	9 (27%)
Median age (IQR)	69 (59.5–75.5)
Median BMI (IQR)	29 (25.6–31)
ASA grade	
I	1
II	27
III	4
IV	0
Neo adjuvant chemoradiotherapy	11 (33%)

The short-term surgical outcomes of the patients who underwent robotic rectal surgery are shown in Table 2. There were no conversions to open or laparoscopy, no anastomotic leaks and no 30-day mortality. Median operative time was 331 min (IQR 249–372), blood loss 20 ml (IQR 20–45) and length of stay 6.5 days (IQR 4–8). All circumferential resection margins were reported clear (R0).

**Table 2:** Peri-operative and post-operative outcomes

	Total (*n* = 33)
Procedure	
Anterior resection	29 (88%)
Abdominoperineal excision	4 (12%)
Conversion to open	0
Median operative time in minutes (IQR)	331 (249–372)
Median blood loss in mls (IQR)	20 (20–45)
Median length of stay in days (IQR)	6.5 (4–8)
30-day readmission	1 (3%)
30-day re-operation	1 (3%)
Anastomotic leak	0
30-day mortality	0
R0	33 (100%)

There was only one 30-day readmission (3%), a patient who presented 4 weeks after her operation with signs of small bowel obstruction; she did not require any surgery and settled with conservative management.

One patient returned to theatre after developing a chyle leak discharging from the pelvic drain. Initially the drain contents were thought to be secondary to a gastric or duodenal perforation; hence a diagnostic laparoscopy was performed.

## Discussion

The new robotic platform by Intuitive Surgical comes with several technological advances that help overcome many of the difficulties encountered in robotic rectal surgery by its predecessors. A redesigned patient cart with new overhead instrument arm architecture coupled with a laser target system makes docking much easier, quicker and enables multi-quadrant surgery without having to reposition the patient cart. New thinner longer arms equipped with newly designed joints offer a greater range of freedom of motion with less arm clashing. New integrated technologies such as the table motion allow for the table to be moved while the patient cart is still docked, making it ideal for multi-quadrant totally robotic single docking surgery.

To facilitate training and enable reproducibility a standardised modular approach to surgery is required. In this technical note, we describe such an approach for robotic rectal surgery with the da Vinci Xi system and demonstrate its safety and feasibility.

## Electronic supplementary material

Below is the link to the electronic supplementary material.

Supplementary material 1 (WMV 8,69,291 kb)
